# Prognostic Role of Measurement of Renal Resistive Index in Systemic Sclerosis

**DOI:** 10.31138/mjr.32.4.345

**Published:** 2021-12-27

**Authors:** Shefali Khanna Sharma, Arghya Chattopadhyay, Siddharth Jain, Chitra Raj Sharma, Debashish Mishra, Manish Rathi, Mahesh Prakash, Sanjay Jain

**Affiliations:** 1 Clinical Immunology and Rheumatology Division;; 2 Department of Internal Medicine;; 3 Department of Nephrology;; 4 Department of Radiodiagnosis, Postgraduate Institute of Medical Education and Research, Chandigarh, India

**Keywords:** vasculopathy, systemic sclerosis, renal resistive index, renal doppler indices, pulmonary hypertension, digital ulcers, proteinuria

## Abstract

**Objective:**

The spectrum of vascular involvement in systemic sclerosis (SSc) includes digital ulcers, gangrene, Raynaud’s phenomenon, renovascular disease, and pulmonary hypertension (PH). Recognition of markers of subclinical vascular disease in SSc is an area of active research, but such studies are limited. This study assesses the role of measurement of the renal resistive index (RRI) as an early marker of renal and systemic vasculopathy. It is a step forward towards examining the possibility of a “unified vascular phenotype’ in SSc.

**Methods:**

In this single-centre prospective study, RRI was calculated for SSc patients >18 years age. Elevated RRI (>0.7) was correlated with renal function (eGFR and proteinuria) and systemic vasculopathy manifestations like digital ulcers, digital infarcts, and PH.

**Results:**

A total of 73 patients with mean (SD) age 41.8(10.9) years were included. Mean (SD) RRI in the right and left renal artery was 0.65(0.08) and 0.66(0.07), respectively. 16 (21.9%) patients had elevated RRI (>0.7). A strong negative correlation was noted between elevated RRI and eGFR (r= −0.96, p=0.03). The percentage of patients with overt proteinuria was higher in the group with elevated RRI (20% versus 7%) (p=0.16). Similarly, digital ulcers (56% vs 33%) and digital pitting (50% vs 35%.) were numerically higher in the group with raised RRI, although statistical significance was not reached because of small numbers (p=0.09 and 0.28, respectively). No correlation of RRI with PH was identified.

**Conclusion:**

RRI correlates well with asymptomatic renal dysfunction and holds promise in the assessment of systemic vasculopathy. However, validation in studies with a larger sample size is needed.

## KEY MESSAGES:

1. Renal resistive index (RRI) correlates well with subclinical renal disease in systemic sclerosis.
2. RRI is a promising non-invasive surrogate for assessment of systemic vasculopathy in systemic sclerosis.

## INTRODUCTION

Systemic sclerosis (SSc) is a chronic multisystem autoimmune disorder typified by inflammation, vasculopathy and fibrosis occurring concurrently in skin and internal organs.^1^ The spectrum of vascular involvement in SSc is diverse, leading to varied clinical manifestations like Raynaud’s phenomenon, digital ulcers or gangrene, pulmonary hypertension (PH), and renal disease. However, once clinically evident vasculopathy develops, the disease process is often irreversible. Thus, recognizing markers of subclinical vascular disease is a research interest of priority in SSc. Studies to this end have been limited. Sub-nephrotic proteinuria and renal doppler indices hold promise in this regard. Renal resistive index (RRI) estimated by doppler has established utility in ascertaining renovascular damage in non-obstructive renal diseases, where it correlates with renal function and biopsy findings.^2^ Preliminary work in RRI in SSc has shown a correlation of raised RRI with longer disease duration,^3^ advanced nailfold capillary changes4 and a higher likelihood of development of digital ulcers.^5^ However, its correlation with renal function (creatinine clearance) has been conflicting.^3,4,6^ Its correlation with PH has never been evaluated. Ethnic differences have also been proposed.

The present study was conducted to calculate RRI in our Indian SSc patient cohort and assess its prognostic utility through correlation with renal function and features of systemic vasculopathy like digital ulceration, gangrene and PH.

## MATERIALS AND METHODS

This single-centre prospective study recruited consecutive patients of SSc aged ≥18 years meeting the American College of Rheumatology/European League Against Rheumatism 2013 classification criteria^7^ from Rheumatology Clinic of the Postgraduate Institute of Medical Education and Research, Chandigarh, a tertiary care institution in North India, between July 2015 to October 2016. Patients with overlap syndromes, diabetes mellitus, pre-existing renal disease, acute febrile illness and pregnancy were excluded. Ultrasound doppler of the kidneys was done using a Philips 14–22 scanner (Philips India Limited, Haryana, India) using a curvilinear probe. RRI was calculated adjacent to medullary pyramids at the level of the interlobar or arcuate arteries.^8^ RRI>0.70 was considered elevated.^6,8^ Estimation of glomerular filtration rate (eGFR) was done using the Chronic Kidney Disease Epidemiology Collaboration (CKD-EPI) equation. A diagnosis of pulmonary hypertension (PH) was made based on clinical and transthoracic echocardiographic findings. Microalbuminuria was defined as 24-hour-urine-albumin 30–300mg/day; overt proteinuria was defined as 24-hour-urine-protein >300 mg/day.

The study was approved by the Institutional Ethics Committee and the principles of the 1964 Helsinki declaration were adhered to.

### Statistical analysis

Statistical analysis was done using the Statistical Package for Social Sciences (SPSS Inc., Chicago, IL, version 20). Continuous variables were summarized as mean and standard deviation. Categorical variables were summarised as frequencies and proportions. Kolmogorov Smirnov test was used for normality testing. T-test and Mann Whitney U test were used for comparison of means in normally distributed and skewed data respectively. Proportions were compared using Chi square or Fisher’s exact test as applicable. Pearson or Spearman correlation coefficients were calculated for ascertaining correlation between variables. All statistical tests were two-sided and a P value ≤ 0.05 was considered statistically significant.

## RESULTS

Total 84 patients were screened, of which 11 were excluded due to poor breath holding or unwillingness to participate in the study. 73 patients were included in the final analysis. 67 (92%) of them were females. Mean (SD) age of the population was 41.8 (10.9) years. Mean (SD) duration of illness was 6.6 (5.1) years. Mean (SD) BMI was 22.7 (3.6) kg/m^2^. Baseline demographic and clinical features were similar in SSc patients with elevated RRI versus normal RRI (**Table 1**). ANA was done in 84 patients and was positive in 98.8% of patients (83 out of 84). The most common pattern observed in the present study was AC 29 (26.2%).^9^

**Table 1.** Comparative demography, clinical features and treatment used amongst patients with normal vs. raised RRI.

	**Normal (n=57)**	**Increased RRI (n=16)**	**P value**
Age	40.491 ±10.08	46.43 ±12.81	0.0540
Female	53 (93%)	15 (93.8%)	1.000
Duration of illness	6.930 ±5.68	5.438 ± 3.82	0.324
BMI	22.66 ±3.61	22.731 ± 3.55	0.945
Dysphagia	22 (38.6%)	7 (43%)	0.7521
Dyspnoea	38(66.7%)	9 (56%)	0.4329
Skin	55 (96.5%)	15 (93.8%)	0.6323
Sclerodactyly	42 (73.7%)	13 (81.3%)	0.5358
Digital infarct	20 (35.1%)	8 (50%)	0.2821
Puffy finger	19 (33.3%)	9 (56.3%)	0.0968
Steroid	43(75.4%)	13 (81.3%)	0.6242
Cyclophosphamide	44 (77.2%)	10 (62.5%)	0.2396
Nifedipine	22 (38.6%)	3(18.8%)	0.1431
Tadalafil	11 (19.3%)	6(37.5%)	0.1307
Steroid	49 (86%)	13 (81.3%)	0.6443

The mean RRI in the right and left renal artery was 0.65±0.08 and 0.66±0.07, respectively. 16 (21.9%) patients had increased RRI (>0.7). 14 (19%) patients had low eGFR (CKD-EPI) (**Table 1**). A strong negative correlation was noted between elevated RRI and eGFR (r=−0.96, p=0.03). However, on multivariate analysis, the significance was lost, likely due to small number of patients in the current cohort. Overt proteinuria was seen in 7 (9.6%) patients and microalbuminuria was noted in 5 (6.8%) patients. Of the seven patients with overt proteinuria, six had normal eGFR. Percentage of patients with overt proteinuria was higher in the group with elevated RRI (20% versus 7%) (p=0.16) (**Table 2**). Data on pulmonary arterial pressures was available for 60 patients in our cohort. Of these, 17 patients (28%) had PH. No correlation of elevated RRI with PH was identified (**Table 2**). Digital ulcers and digital infarcts or pitted scars were seen in 28 (38%) patients each. Both digital ulcers (56% vs 33%) and digital pitted scars (50% vs 35%) were numerically higher in the group with elevated RRI, although statistical significance was not reached (p=0.09 and 0.28 respectively) (**Table 2**).

**Table 2.** Correlation of elevated renal resistive index (RRI) with systemic vasculopathic manifestations, estimated GFR and proteinuria.

**Parameter**	**Renal Resistive Index**		**P-value**

	**Elevated (>0.7)**	**Normal (<0.7)**	
Digital ulcer (%) (n=73)	9/16 (56.3%)	19/57 (33.3%)	0.09

Digital infarct (%) (n=73)	8/16 (50%)	20/57 (35.1%)	0.28

Pulmonary Hypertension (%) (n=60)	4/13 (30.8%)	13/47 (27.7%)	0.95

Proteinuria (%) (n=71)	3/15 (20%)	4/56 (7.1%)	0.16

eGFR (mL/min/1.73 m^2^) (n=73)			0.03
▪ >90	10 (62.5%)	49 (86%)	
▪ 60–89	2 (12.5%)	8 (14%)	
▪ 30–59	2 (12.5%)	0	
▪ <30	2 (12.5%)	0	

eGFR: estimated glomerular filtration rate.

## DISCUSSION

Vasculopathy is a vital pathogenic process in SSc, responsible for varied clinical manifestations like renal dysfunction, pulmonary hypertension, Raynaud’s phenomenon, cutaneous ulcers and digital gangrene. Renal dysfunction and/or gross proteinuria in SSc occurs in the setting of advanced renal vasculopathy, usually when the underlying disease process has crossed the stage of reversibility. Identification of markers of subclinical disease thus assumes great importance in SSc. Studies to this end have been limited so far. RRI, a non-invasive tool to assess intrarenal vasculature and tubulointerstitial compartment, offers hope for detection of subclinical renal disease. Owing to shared pathogenetic mechanisms, the authors believe that ascertainment of vasculopathic process at one site (renal, in the present study) could hypothetically be a surrogate marker of the extent or severity of vasculopathy at other sites, as assessed in the present study.

Campto et al. found PH in SSc to be associated with renal dysfunction, with a low eGFR leading to a threefold increased risk of mortality in SSc-PH.^10^ Whether this association is by virtue of cardiorenal syndrome produced by PH-related cardiac dysfunction, or is due to a common underlying pathogenetic mechanism (ie, vasculopathy) is unknown. This study is probably the first attempt to look for an association between PH and sub-clinical renal disease assessed by RRI. No correlation of PH with raised RRI was noted. However, limitation of the current study was PH assessed by echocardiography only. The etiopathogenesis of PH in SSc is multifactorial - vasculopathy of small pulmonary arteries (Group 1), ILD (Group 3), myocardial fibrosis (Group 2) or pulmonary veno-occlusive disease.^11^ The lack of correlation of PH with RRI (which assesses only the vasculopathic component of PH) could be reflective of this multifactorial causation, as well as the inherent limitations of estimation of PH by echocardiography.^12^

Conflicting data exist regarding correlation of RRI with renal function in SSc. No correlation with creatinine clearance was noted in an initial study by Rivolta et al.^3^ however subsequent studies have demonstrated a negative correlation between GFR and renal doppler indices.^4,6,13^ In the present study, a strong negative correlation was noted. In a follow-up study of 4.1 years, no decline in renal function was noted despite increased intraarterial stiffness as measured by renal doppler indices.^6^ RRI might thus be an early marker of subclinical renal involvement, even before the appearance of proteinuria.

In our cohort, patients with raised RRI had a higher prevalence of digital ulceration and pitting reflecting an underlying systemic vasculopathy, although statistical significance was not reached, because of the small sample size. This echoes with the results of two previously published studies.^4,5^ Nailfold capillaroscopy changes were not assessed in the present study.

Asymptomatic renal function impairment (proteinuria or low eGFR) is seen variably in 10–55% patients of SSc.^14^ However, the significance of proteinuria and microalbuminuria is uncertain, with some studies calling them “benign and non-progressive”, whilst others believe them to be a reflection of renal functional reserve.^14^ Seiberlich et al. noted intermediate-weight proteinuria in SSc, which correlated with increased blood pressure, gastrointestinal and pulmonary involvement.^15^ Attempts at correlation between proteinuria and RRI in SSc have been minimalistic. In the present study, the proportion of patients with overt proteinuria was higher in the group with elevated RRI (20% versus 7.1%), although statistical significance was not reached because of small numbers. The prevalence of proteinuria in our study was comparatively low, due to inclusion of limited cutaneous SSc, exclusion of diabetes, pregnancy, absence of penicillamine use and ethnic differences in renal involvement in Asian SSc patients.^16^

In a study by Gigante A et al. has shown changes of RRI over time correlated with different vascular complications.^17^ Thus, measurement of RRI holds promise for assessment of subclinical renal and systemic vasculopathy in SSc, and needs to be validated in larger prospective studies.

It is important to note that many hemodynamic, physiological, and disease-related factors influence the intra-renal arterial Doppler waveforms, thus limiting the utility and external validity of renal vascular doppler indices.^8^ The variability produced by these physiological and pathological factors may explain the discrepant direction and strength of correlation seen in different studies.

The strength of the present study lies in its comprehensive attempt at correlation of RRI with most systemic vasculopathic manifestations in patients of SSc. This is the first study to examine correlation between PH and RRI. However, this study has limitations- a relatively small sample size leading to statistically insignificant results, use of echocardiography for PH assessment and unavailability of nailfold capillaroscopy changes.

## CONCLUSION

RRI correlates well with asymptomatic renal dysfunction and is a promising tool for assessment of systemic vasculopathy in systemic sclerosis. However, validation in studies with larger sample size is needed.
